# The immune-inflammatory response of oligodendrocytes in a murine model of preterm white matter injury: the role of TLR3 activation

**DOI:** 10.1038/s41419-021-03446-9

**Published:** 2021-02-08

**Authors:** Marta Boccazzi, Juliette Van Steenwinckel, Anne-Laure Schang, Valérie Faivre, Tifenn Le Charpentier, Cindy Bokobza, Zsolt Csaba, Claudia Verderio, Marta Fumagalli, Shyamala Mani, Pierre Gressens

**Affiliations:** 1 Université de Paris, Inserm UMR 1141 NeuroDiderot, F-75019 Paris, France; 2 PremUP, F-75006 Paris, France; 3 Université de Paris, Inserm UMR 1153, Centre de recherche en Epidémiologie et Statistiques (CRESS), Equipe HERA, Paris, France; 4 CNR Institute of Neuroscience, via Vanvitelli 32, 20129 Milan, Italy; 5 Department of Pharmacological and Biomolecular Sciences, Università degli Studi di Milano, via Balzaretti 9, 20133 Milan, Italy; 6 Curadev Pharma Pvt. Ltd, Noida, India; 7 Centre for the Developing Brain, Division of Imaging Sciences and Biomedical Engineering, King’s College London, King’s Health Partners, St. Thomas’ Hospital, London, SE1 7EH UK; 8 Present Address: Department of Pharmacological and Biomolecular Sciences, Università degli Studi di Milano, via Balzaretti 9, 20133 Milan, Italy

**Keywords:** Encephalopathy, Oligodendrocyte

## Abstract

A leading cause of preterm birth is the exposure to systemic inflammation (maternal/fetal infection), which leads to neuroinflammation and white matter injury (WMI). A wide range of cytokines and chemokines are expressed and upregulated in oligodendrocytes (OLs) in response to inflammation and numerous reports show that OLs express several receptors for immune related molecules, which enable them to sense inflammation and to react. However, the role of OL immune response in WMI is unclear. Here, we focus our study on toll-like receptor-3 (TLR3) that is activated by double-strand RNA (dsRNA) and promotes neuroinflammation. Despite its importance, its expression and role in OLs remain unclear. We used an in vivo mouse model, which mimics inflammation-mediated WMI of preterm born infants consisting of intraperitoneal injection of IL-1β from P1 to P5. In the IL-1β-treated animals, we observed the upregulation of *Tlr3, IL-1β, IFN-β, Ccl2*, and *Cxcl10* in both PDGFRα+ and O4+ sorted cells. This upregulation was higher in O4+ immature OLs (immOLs) as compared to PDGFRα+ OL precursor cells (OPCs), suggesting a different sensitivity to neuroinflammation. These observations were confirmed in OL primary cultures: cells treated with TLR3 agonist Poly(I:C) during differentiation showed a stronger upregulation of *Ccl2* and *Cxcl10* compared to cells treated during proliferation and led to decreased expression of myelin genes. Finally, OLs were able to modulate microglia phenotype and function depending on their maturation state as assessed by qPCR using validated markers for immunomodulatory, proinflammatory, and anti-inflammatory phenotypes and by phagocytosis and morphological analysis. These results show that during inflammation the response of OLs can play an autonomous role in blocking their own differentiation: in addition, the immune activation of OLs may play an important role in shaping the response of microglia during inflammation.

## Introduction

Maternal/foetal infection can perturb development increasing the risk for permanent neurological and neuropsychological disorders^1,2^. Sterile systemic inflammation induced by IL-1β injections in newborn mouse results in hypomyelination and is a model for diffuse white matter injury (WMI) observed in human preterm infants^3^. Myelination requires proliferation of oligodendrocyte precursor cells (OPCs) and maturation of premyelinating oligodendrocytes (OLs) into myelinating OLs, a process blocked in this model^4^. Although previous studies have shown a causal link between microglial activation and myelination defects^5^ how systemic inflammation leads to a block in OL maturation is not completely understood.

Innate immune response is the first line of defense against infection and plays an important role in sterile inflammation through the production of type I IFNs and proinflammatory cytokines^6^. Toll-like receptor-3 (TLR3) is a member of the TLR family of innate immune response receptors and has a unique expression pattern and subcellular localization^7,8^. Aside from double-strand RNA (dsRNA) derived from infectious agents^9^, it can be activated by damaged cells caused by sterile inflammation^10–12^. TLR3 expression in the neonatal brain has been demonstrated^13^ and its activation during development results in several cellular and behavioral abnormalities^14–17^. Alterations in TLR3 have been reported in WMI, including receptor upregulation in the lesion area and in the adjacent WM regions, altered localization in neurons and increased expression in reactive astrocytes^13,18^.

Here we studied whether TLR3 in OLs could contribute to maturation block during perinatal inflammation. We tested whether inflammation has a differential effect on different populations of immature OLs^3^ both in vivo and following TLR3 activation in vitro. Finally, we tested whether immune response of OLs can modulate microglial activity.

## Materials and methods

### In vivo IL-1β administration

Experimental protocols were approved by the institutional guidelines of the Institut National de la Santé et de la Recherche Scientifique (Inserm, France) and met the guidelines for the United States Public Health Service’s Policy on Humane Care and Use of Laboratory Animals (NIH, Bethesda, Maryland, USA). The protocol was approved by the Bichat-Robert Debre ethical committee under the reference 2011-14/ 676-0053. OF1 strain pregnant female were purchased from Charles River (L’Arbresle, France) and male pups were randomly assigned to treatment or control group. IL-1β exposure was carried out as previously described^3^. In brief, mice received twice a day from postnatal day 1 (P1) to P4 and once on P5 a 5-μL intraperitoneal injection of 10 ng/g/injection recombinant mouse IL-1β in phosphate buffered saline (PBS; R&D Systems, Minneapolis, MN, USA) or PBS alone. Animals were sacrificed 4 h after the morning injection of IL-1β at P3, P5, and P10.

### PDGFRα**+** and O4**+** magnetic-activated cell sorting

**Fig. 1:**
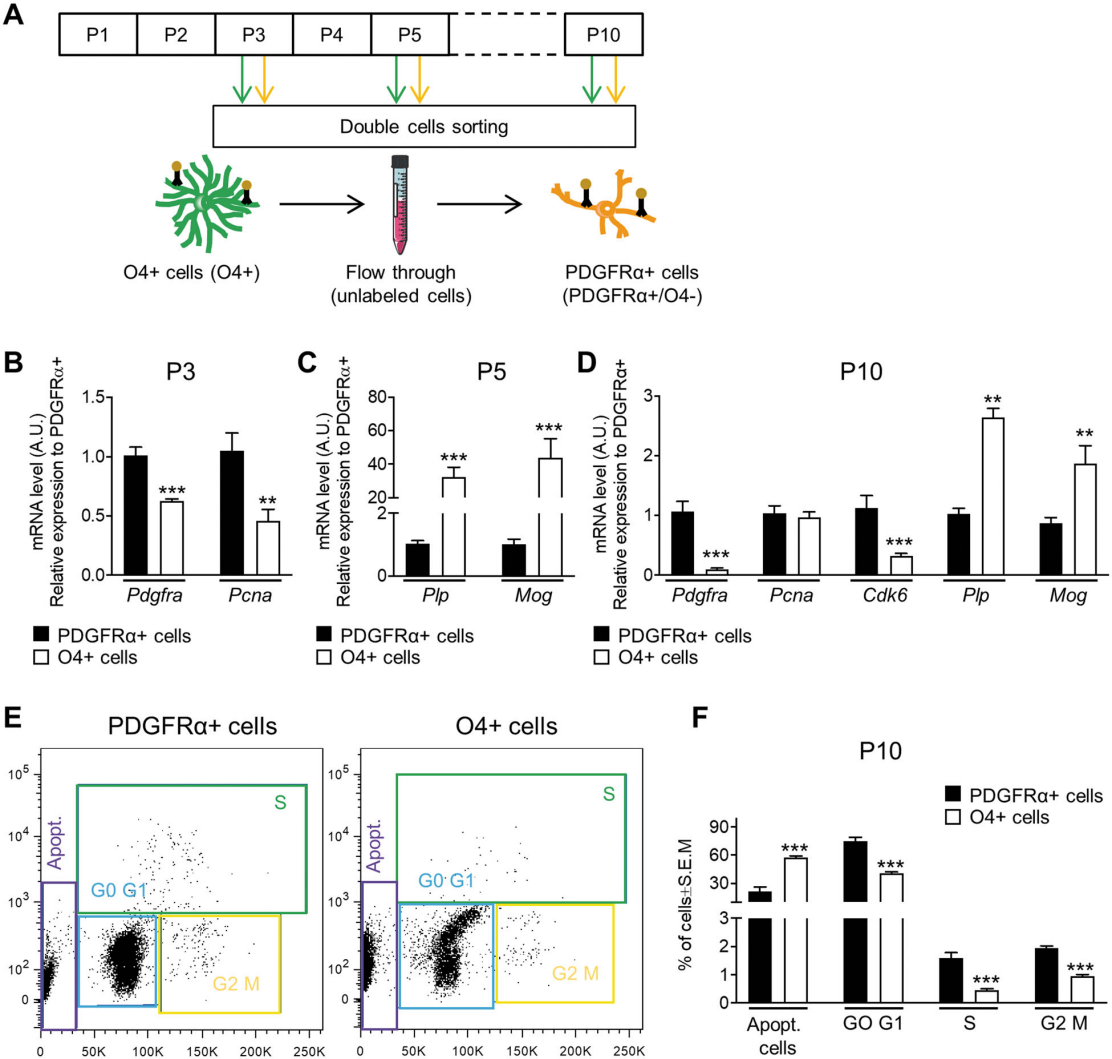
PDGFRα+ and O4+ sorted cells have a different proliferative and differentiative potential. **A** Schematic representation of the sequential isolation of O4+ and PDGFRα+ cells by magnetic bead separation (MACS) from control male mice at P3, P5, and P10. **B**–**D** qPCR analysis of different markers of cell proliferation (*Pcna* and *Cdk6*) and differentiation (*Pdgfrα, Mog, Plp*) in PDGFRα+ and O4^+^ sorted cells at **B** P3, **C** P5, and **D** P10 (data are mean ± SEM, *n* = 6 groups/condition at P3 and P10 and *n* = 18 groups/condition from three independent experiments at P5, **P < 0.01 or ***P < 0.001 Unpaired *t*-Test). **E**, **F** Flow cytometry plots and analyses of the percentages of cells at the different phase of the cell cycle (data are mean ± SEM, *n* = 6 groups/condition, ***p < 0.001, Unpaired *t*-test).

At P3, P5, and P10, brains were collected for cell dissociation and for PDGFRα-positive and O4-positive cell enrichment using a magnetic coupled antibody (MACS, Miltenyi Biotec, Bergisch Gladbach, Germany), as previously described^4,19^ and according to the manufacturer’s protocol. In brief, pooled brains (*n* = 4 at P3, *n* = 3 at P5, and *n* = 2 at P10) were dissociated using the Papain Neural Tissue Dissociation Kit. Brain homogenate cells were first incubated with the anti-O4 microBeads antibody and O4+ cells were isolated. O4+ cells are both PDGFRα- and PDGFRα+ (but hereafter referred as O4+ cells). Flow through containing the O4− fraction were incubated with the anti-PDGFRα microBeads antibody; thus, these cells were PDGFRα+/O4− (referred as PDGFRα+; Fig. 1A).

### Flow cytometry

Cell cycle was analyzed by 5-ethynyl-2-deoxyuridine (EdU) incorporation and DNA content measurement using 647 EdU Click Proliferation Kit (BD Biosciences, San Diego, CA, USA) and propidium iodide (PI, Sigma, St Louis, MO, USA). EdU was added to sorted cell during 3 h; click reaction and PI staining were performed according to manufacturer recommendations. The gating strategy selected FSC and SSC singlets, followed by selection of cells in “G0/G1”, “S”, and “G2/M” phases. Cells with low DNA content were defined as apoptotic.

### Primary cultures

Brains from P5 pups were dissociated with Papain Neural Tissue Dissociation Kit^4,20^ and OPCs or microglia were enriched by MACS using the anti-PDGFRα or anti-CD11b MicroBeads, respectively.

### OPCs primary culture

After cell sorting, OPCs were cultured as previously described^21,22^. Cells were treated with the TLR3 agonist Poly(I:C) (PIC, 50 ug/ml; Sigma) and kept in proliferation or differentiation medium for additional 72 h (72 h). In selected experiments, cells were treated with IFNγ (either 8 or 16 ng/mL; Miltenyi).

To assess proliferation, cells were maintained in proliferation or differentiation condition for 72 h and incubated with 5 µM EdU for the last 16 h at 37 °C. EdU incorporation was detected with the Click-iT EdU Alexa Fluor-594 Imaging Kit according to manufacturer’s protocol.

### Multiplex cytokine/chemokine assay

Proliferation and differentiation medium harvested from OL culture after 72 h was centrifuged briefly to remove particulates (600 × *g* for 10 min). CCL2 (MCP- 1), CCL3 (MIP-1a) CCL5 (RANTES), and CCL11 (Eotaxin) levels were measured using a Bio-plex 200 according to the manufacturer’s instructions (Biorad laboratories, Marnes la Coquette, France). All samples were run in duplicates and data were analyzed with the Bio-Plex Manager software.

### Microglia primary culture

After cell sorting, microglia were cultured as previously described^5^. For medium conditioning, proliferation and differentiation medium was collected from OL culture after 24 h or 72 h and spun at 600 × *g* for 10 min to remove cell debris. 0.8 mL (27%) of conditioned medium (CM) was added to 2.2 mL of microglia medium and 1 mL added to each well for stimulation. For PIC condition, PIC was added at the concentration present in the CM directly to microglia media (13.3 μg/mL corresponding to 27% of 50 μg/mL) and at two different dilutions: 4.4 μg/mL and 1.33 μg/mL. Twenty hour after stimulation RNA was extracted.

To evaluate phagocytosis, microglia were plated in μ-Slide 8-Well Glass Bottom chamber slides (Ibidi, BioValley; 100,000 cells/well). Fluorescent latex beads were activated by incubation with 50% FBS for one hour at 37 degrees with frequent agitation. 100 beads/cells were added to microglia after 21 h of stimulation with PIC or the CM. After 3 h cells were washed twice with PBS and then fixed with 4% PFA.

### Immunocytochemistry

Primary cells were fixed (in 4% PFA) and immunolabelled as previously described^23^. Mouse anti-NG2 (1:200; ab50009, Abcam, France), rabbit anti-GPR17 (1:100, custom antibody produced by PRIMM, Milan, Italy), mouse anti-O4 (1:100, MAB 1326, Bio-Techne SAS, France) mouse anti-MBP (1:200, MAB382, Merck Chimie SAS, France), and goat anti-Iba1 (1:500, abcam) primary antibodies were used. Fluorescent anti-goat, anti-rabbit or anti-mouse IgG (all 1:600, 1 h at RT; Life Technologies, USA) secondary antibodies were used. Double labeling with anti-O4 was performed using detergent-free buffers. Nuclei were counterstained with DAPI (Sigma–Aldrich). Images were acquired using a fluorescent microscope (Nikon Eclipse Ti‐E) and all the analyses were performed with the ImageJ (NIH). For oligodendrocytes, in each coverslip the total number of DAPI+ nuclei and the number of MBP+ cells were determined in 20–25 optical fields chosen according to a pre-established scheme. Measurements of the total surface area and the surface area covered by MBP immunostaining were performed using an appropriate macro design.

Fluorescent microbeads were quantified on IBA1+ cells. Results were expressed as the percentage of phagocytic cells (number of cell with beads/number of total IBA1+ cells). For microglial morphological analysis between 257 and 349 single cells/group have been analyzed and on the basis of IBA1 immunostaining cells area, cells perimeter, and cells sphericity using the relation 4π × (area/perimeter2)^24^ have been measured.

### RNA extraction and qPCR

Preparation of samples for qRT- PCR, primer design, and PCR protocol were similar to that previously described^19,20^. Primer sequences are given in Supplementary Table 1. The relative expression of genes of interest was determined relative to expression of the reference gene, Ribosomal Protein L13 (*Rpl13*). Analyses were performed with the Biorad CFX manager 2.1 software and a relative quantification approach was used, according to the 2-ddCT method^25^.

### Statistical analysis

All data are reported as means ± SEM. Statistical analysis of all data was performed using GraphPad PRISM version 6.0 (San Diego, CA). Data were tested for normality using the Kolmogorov–Smirnov and Bartlet test or F-test were used to evaluate that variances were equal across groups. In case of single comparisons, the Student’s *t*-test was applied whereas for more than two groups a one-way analysis of variance (ANOVA) followed by Bonferroni post-hoc multiple test was used. For all analyses, statistical significance is denoted as *P < 0.05, **P < 0.01, or ***P < 0.001. For in vivo experiments there were at least six biological replicates at each timepoint whereas in vitro experiments are performed at least in triplicate to provide adequate statistical power. No power analysis was performed. No data were excluded. No blinding was done.

## Results

### PDGFRα+ and O4+ cell populations have different proliferation and differentiation properties

Sequential isolation of O4+ and PDGFRα+ /O4− cells by MACS at P3, P5, and P10 (Fig. 1A). At P3, PDGFRα+ cells had a higher expression of proliferating cell nuclear antigen (*Pcna* mRNA) than O4+ cells (Fig. 1B). The O4+ population, already committed towards differentiation, showed lower *Pcna* and *Pdgfrα* expression (Fig. 1B). At P5 the O4+ population exhibited higher mRNA expression for myelin genes (Fig. 1C) and at P10 the expression of *Pdgfrα* was strongly reduced (Fig. 1D). Of note, at this stage no difference in *Pcna* expression was detected between the two cell populations. However, the level of *Cdk6* mRNA, whose downregulation is important for differentiation^26^, was significantly lower in O4+ cells (Fig. 1D). Reduced percentage of O4+ cells in all phases of the cell cycle was confirmed by FACS analysis (Fig. 1E, F). This was associated with a higher percentage of apoptotic O4+ cells, described previously^27^.

### *Tlr3* receptor expression is regulated in oligodendrocytes during development and induced by neuroinflammation

**Fig. 2:**
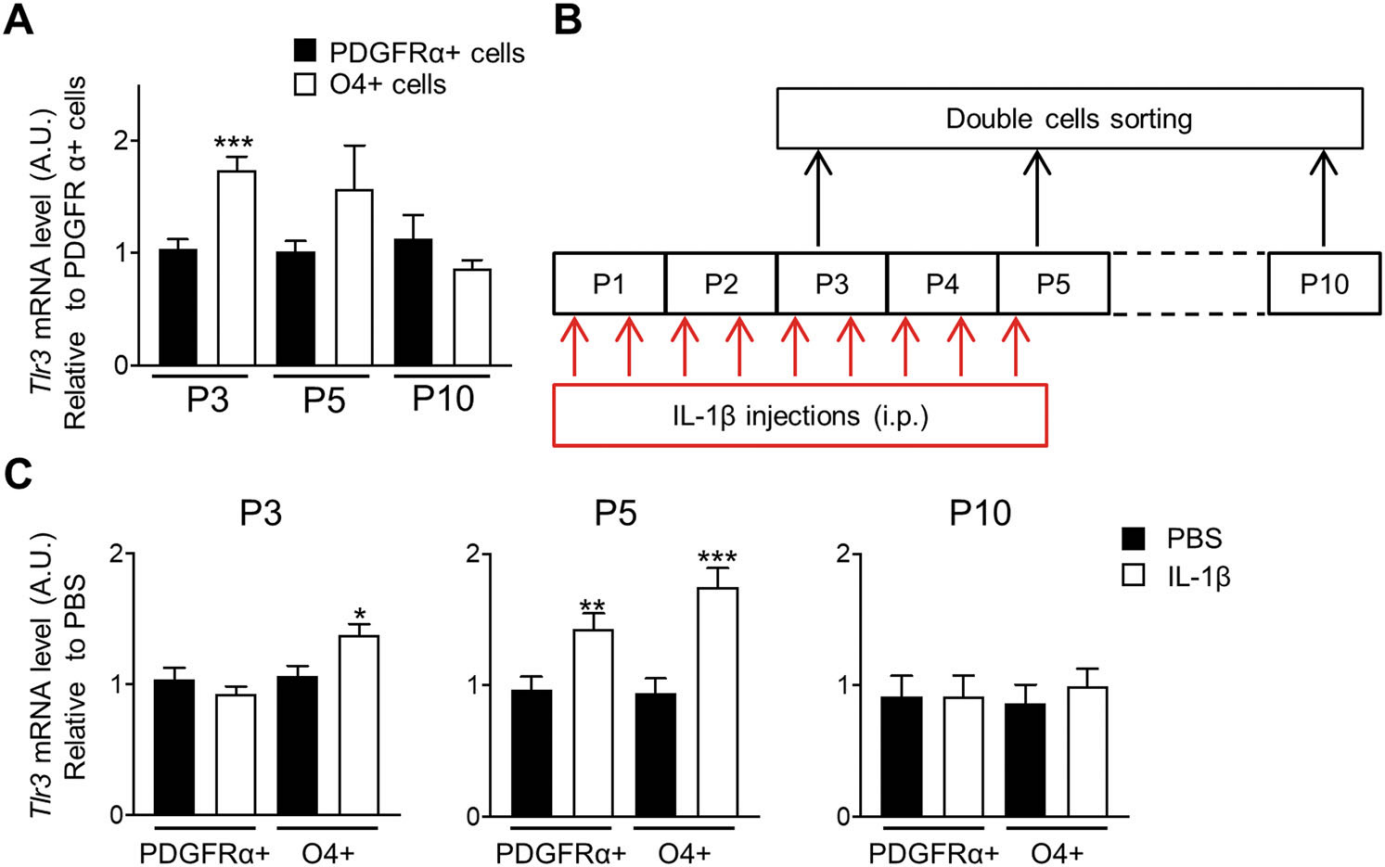
*Tlr3* expression is differentially regulated in PDGFRα+ OPCs and O4+ immOLs during development and is induced after IL-1β treatment. **A** qPCR analysis of *Tlr3* expression in PDGFRα+ OPCs and O4+ immOLs at different postnatal days (P3, P5, and P10) form control male mice brain (data are mean ± SEM, *n* = 10 groups/condition from two independent experiments for P3 and P10 and *n* = 17 groups/condition from three independent experiments at P5, ***p < 0.001 Unpaired *t*-Test). **B** Experimental scheme. **C** *Tlr3* expression assessed by qPCR in control and IL-1β treated mice in PDGFRα+ and O4+ sorted cells (data are mean ± SEM, *n* = 10 groups/condition from 2 independent experiments at P3 and P10, *n* = 18 from 3 independent experiments at P5, *P < 0.05, **P < 0.01, ***P < 0.001 Unpaired *t*-test).

Regulation of *Tlr3* in naïve mice during development was characterized by qRT-PCR. At P3, *Tlr3* expression was increased by 1.7 fold in O4+ cells compared to PDGFRα+ cells, and the trend for higher expression persisted at P5. No difference in *Tlr3* mRNA levels was seen at P10 (Fig. 2A). Next, modulation of *Tlr3* in OLs during perinatal inflammation was studied (Fig. 2B, C). At P3, IL-1β injection increased the mRNA level of *Tlr3* in O4+ cells but not in the PDGFRα+ cells. At P5, IL-1β was also capable of inducing *Tlr3* expression in PDGFRα+ cells. At P10, no further regulation of *Tlr3* mRNA was seen (Fig. 2C). As shown previously^4^, O4+ cells isolated from IL-1β animals showed reduced expression of myelin genes compared to control (Supplementary Fig. 1). Taken together these results indicate that O4+ cells display higher levels of *Tlr3* transcript compared to PDGFRα+ cells and selectively upregulate *Tlr3* expression in response to IL-1β early in development. However, by P5, the PDGFRα+ cells also increased their *Tlr3* expression in response to IL-1β.

### In response to neuroinflammation OLs upregulate expression of innate immunity related chemokines and cytokines

**Fig. 3:**
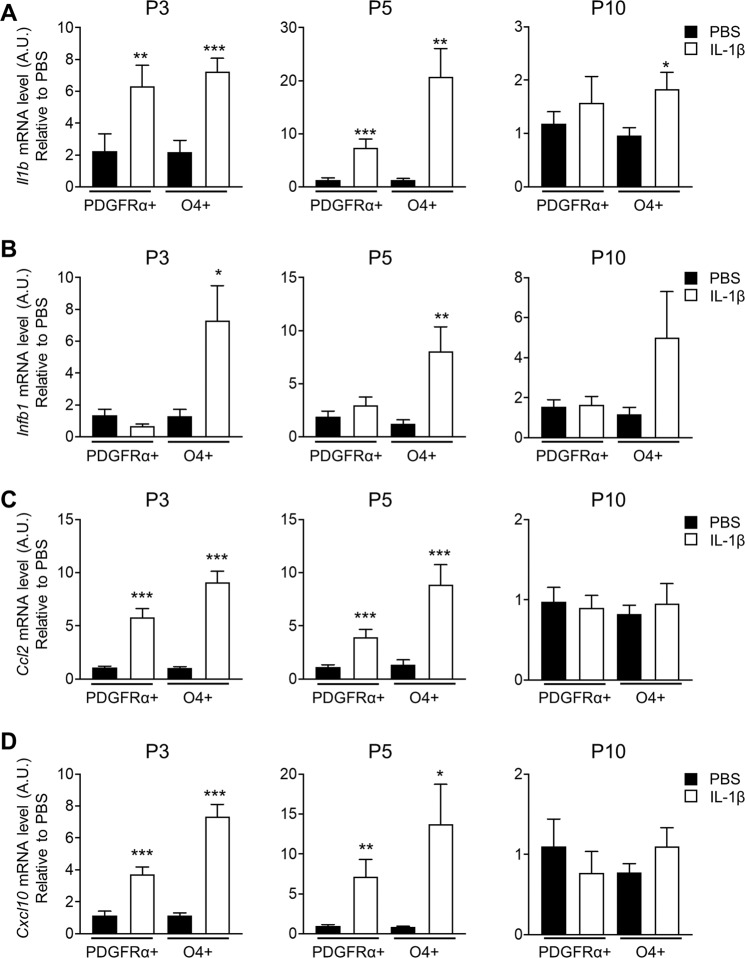
Upon neuroinflammation the PDGFRα+ OPCs and O4+ immOLs upregulate the expression of innate immunity related chemokines and cytokines. **A–D** Expression level of *Il1b* (**A**), *Ifnb1* (**B**), *Ccl2* (**C**), and *Cxcl10* (**D**) assessed by qPCR in PDGFRα+ OPCs and O4+ immOLs MACS-sorted from the brain of PBS and IL-1β-treated mice at P3, P5, and P10 (data are mean ± SEM, *n* = 12 groups/condition from 2 independent experiments for P3 and *n* = 18 groups/condition from three independent experiments for P5 and P10, *P < 0.05, **P < 0.01, ***P < 0.001 Unpaired *t*-test).

Since OLs are capable of influencing an on-going inflammatory response^28^, we assessed whether PDGFRα+ and O4+ cells differed in their ability to upregulate factors associated with an innate immune response (Fig. 3). IL-1β and type I interferons have a pivotal role in innate immunity and CCL2 and CXCL10 play a key role in immune cells recruitment^29^. In response to IL-1β injections, PDGFRα+ and O4+ cells upregulated *Il1b* mRNA at P3 and P5 but only O4+ cells did so at P10 (Fig. 3A). Only O4+ cells responded to IL-1β with an increase in *Ifnb1* expression at P3 and P5, and showed a tendency, although not statistically significant, to maintain elevated *Ifnb1* levels at P10 (Fig. 3B). mRNA for *Ccl2* and *Cxcl10* (Fig. 3C, D) was increased at P3 and P5 in PDGFRα+ and O4+ cells; however, the increase in O4+ cells was greater than that seen for the PDGFRα+ cells. No upregulation was observed at P10. Together, these results suggest that the O4+ cell population has a stronger capacity to upregulate the expression of several inflammatory genes than PDGFRα+ cells. Furthermore, in both cell populations, the immune response occurred only during the time of inflammatory challenge.

### TLR3 activation by Poly(I:C) during differentiation in vitro recapitulates observations in vivo

**Fig. 4:**
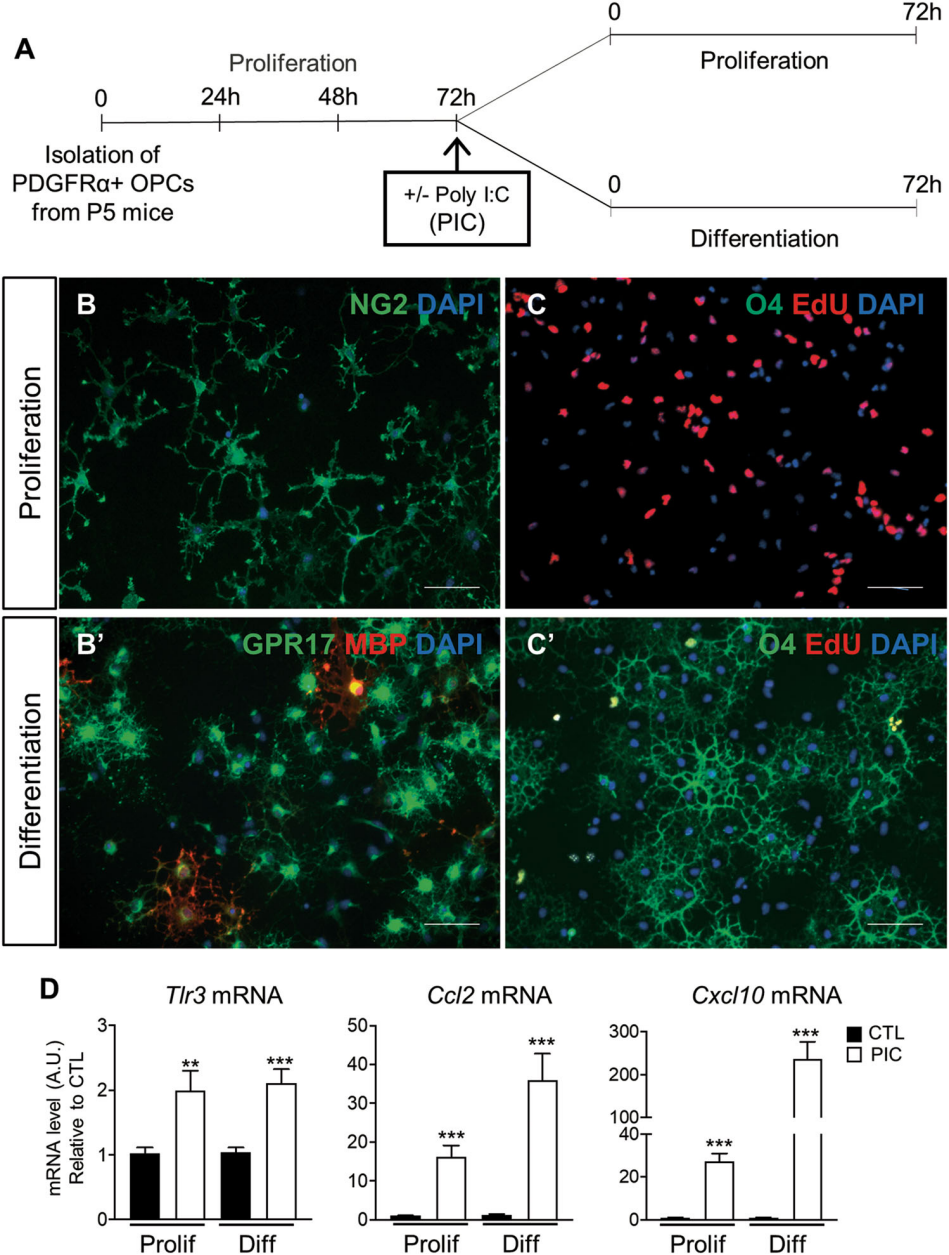
In vitro treatment with TLR3 agonist Poly(I:C) induces TLR3 activation both in proliferating and differentiating OLs. **A** Experimental scheme. **B**–**C**’ Immunofluorescence staining for NG2, O4, GPR17, MBP, and EdU of OLs cultured in proliferating (**B**–**C**) or differentiating (**B**’–**C**’) conditions in control medium (scale bar 100 μm). **D** qPCR analysis of *Tlr3*, *Ccl2*, and *Cxcl10* expression in primary OLs treated with Poly(I:C) (PIC) 50 ug/ml for 72 h in proliferation (Prolif) or differentiation (Diff) medium (data are mean ± SEM of three independent experiments performed in triplicate, **P < 0.01, ***P < 0.001 Unpaired *t*-test).

To model the in vivo results and investigate the role of TLR3 we compared the consequences of TLR3 activation in PDGFRα+ cells cultured either in proliferating conditions (containing bFGF and PDGF-AA) or exposed to differentiating conditions (growth factor withdrawal and addition of thyroid hormones; Fig. 4A). Poly(I:C) (PIC), a synthetic analog of dsRNA that is the natural ligand for TLR3 was used to activate the TLR3 receptor^8^. Cells maintained in proliferation condition expressed the OPC marker NG2 and displayed a bipolar morphology^30^; whereas cells shifted to differentiation started to express the immature marker GPR17^23,31^ and the myelin marker MBP. Moreover, most cells in proliferation media expressed EdU with none of the cells expressing the immature marker O4; whereas, only few cells in differentiation media were EdU+ while most expressed O4 (Fig. 4B, C).

Cultured OPCs and differentiating OLs were treated with PIC and gene expression was monitored. PIC induced *Tlr3* mRNA in both conditions whereas *Ccl2* and *Cxcl10* mRNA induction was greater in differentiated cells compared to cells kept in proliferation condition (Fig. 4D), thereby mimicking our observation in vivo. To test the specificity of this effect to TLR3, we stimulated cultures with IFNγ. Here, both undifferentiated and differentiated cells were able to upregulate *Cxcl10* with no difference in the extent of mRNA expression (Supplementary Fig. 2). In contrast to the in vivo data, we could not reliably detect upregulation of either *Il1b* or *Ifnb1* (data not shown).

**Fig. 5:**
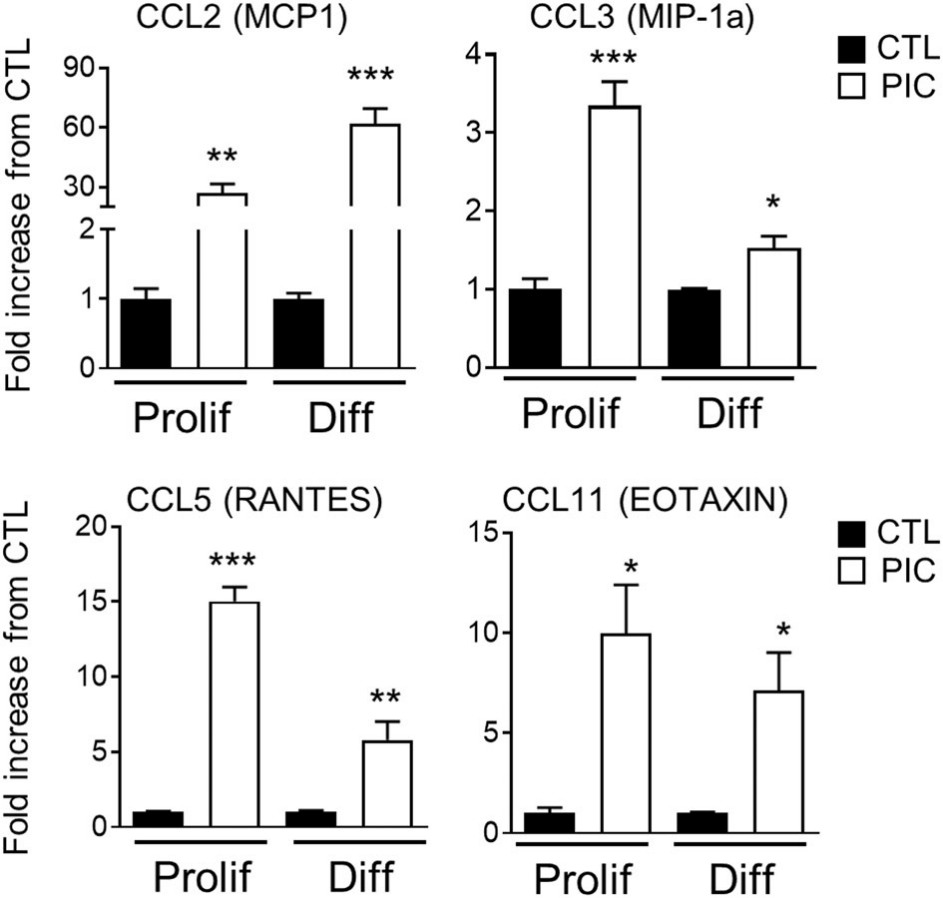
CCL2, CCL3, CCL5, and CCL11 chemokines are differentially expressed at protein level in conditional medium (CM) from proliferating and differentiating OL stimulated with Poly(I:C). The expression of CCL2, CCL3, and CCL5 was measured in the culture media collected from OLs treated with Poly(I:C) (PIC) 50 ug/ml for 72 h in proliferation (Prolif) or differentiation (Diff) conditions (*n* = 3, *P < 0.05, **P < 0.01, and ***P < 0.001 Unpaired *t*-test).

We corroborated the differential increase of Ccl2 mRNA in differentiating cells by showing that the amount of CCL2 protein in the medium derived from OLs maintained in differentiation condition and treated with PIC for 72 h was the highest. This was not the case for CCL3, CCL5, and CCL11 whose levels were either not different or higher in proliferating cells after stimulation with PIC (Fig. 5).

### Addition of Poly(I:C) perturbs differentiation

**Fig. 6:**
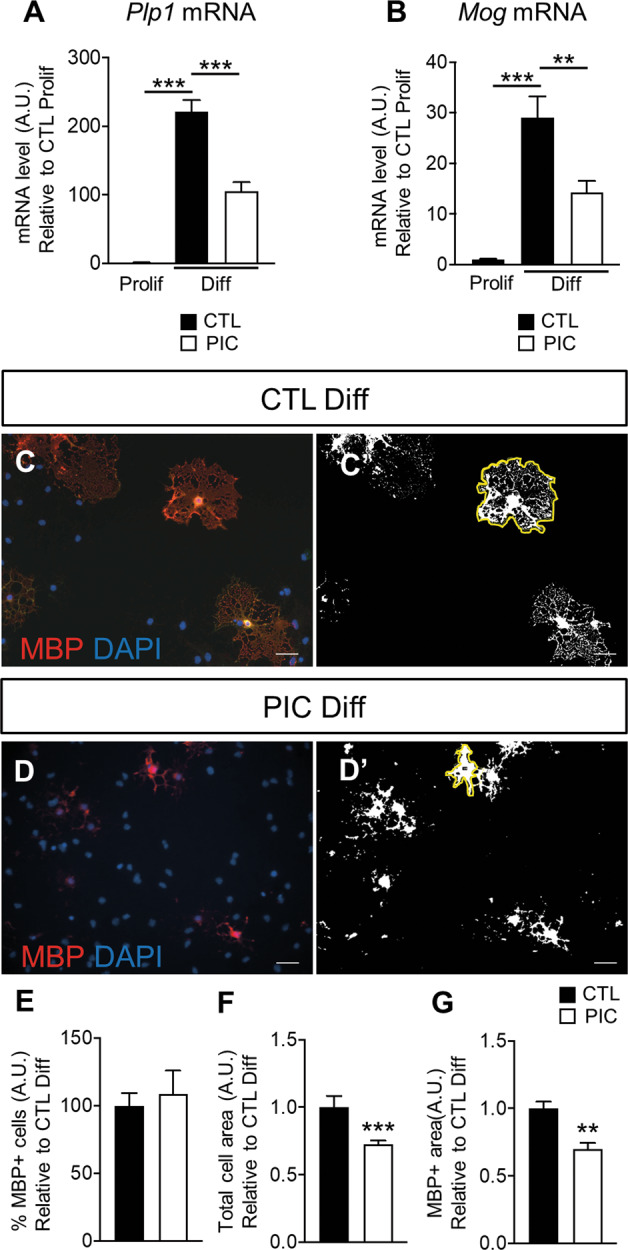
Poly(I:C) treatment during differentiation results in downregulation of myelin genes and defects in reaching the more mature phenotype. **A–B** qPCR analysis of *Mbp* and *Plp1* in primary OLs treated with Poly(I:C) (PIC) 50 ug/ml for 72 h in differentiation (Diff) medium (results are the mean of four independent experiments performed in triplicate ±SEM, **P < 0.01, ***P < 0.001 One-way Anova followed by Bonferroni’s multiple comparison test). **C**–**D**’ Immunofluorescent staining for MBP. To evaluate MBP morphology coloured images (**C**, **D**) have been transformed in binary with ImageJ (**C**’, **D**’). **E**–**G** Histograms reported the percentage of MBP+ cells (**E**), measure of the cell area (**F**, delimited with yellow line in **C**’ and **D**) or of the area covered by MBP immunostaining (**G**, white signal in **C**’ and **D**’; data are mean ± SEM, *n* = 9 coverslips for each condition from three independent experiments, **P < 0.01, ***P < 0.001 Unpaired *t*-test). Scale bar 100 μm.

mRNA encoding myelin genes *Mog* and *Plp1* were downregulated in the presence of PIC (Fig. 6A, B). Furthermore, although there was no difference in the number of MBP-positive cells (Fig. 6E), the area of MBP+ differentiated cells (Fig. 6F) and the area of MBP staining (Fig. 6G) were reduced (respectively, the yellow line and the white signal in Fig. 6C’ and D’), indicating that PIC stimulation limits maturation of pre-oligodendrocytes towards fully mature MBP-positive cells.

### Oligodendrocytes influence microglial activation in vitro

**Fig. 7:**
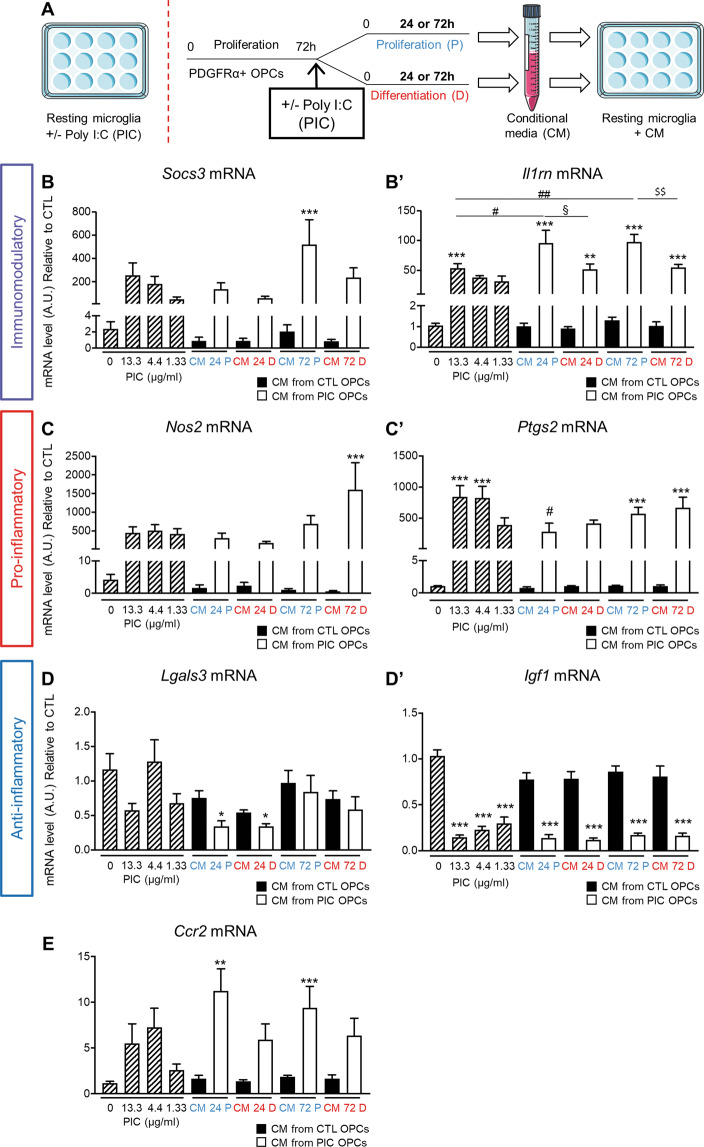
Conditional medium (CM) from proliferating and differentiating OLs regulates microglia differently. **A** Experimental protocol. **B**–**E** qPCR analysis of microglia treated with PIC or conditional medium (CM). Results are the mean of three independent experiments performed in triplicate ±SEM, *P < 0.05, **P < 0.01, and ***P < 0.001 vs CTRL, ^#^*P* < 0.05 PIC 13.3 vs CM 24 P, ^##^*P* < 0.01 PIC 13.3 vs PIC CM 72 P, ^§^*P* < 0.05 PIC CM 24 P vs PIC CM 24D, and ^$$^*P* < 0.01 PIC CM 72 P vs PIC CM 72D, One-way Anova followed by Bonferroni’s multiple comparison test was performed.

Although it is well established that activated microglia can affect OL differentiation the reverse has not been explored much. For this, we took CM from either differentiated OLs or precursors kept in proliferating media in control condition or stimulated with PIC and added 27% of CM to microglia media. As control, microglia was exposed to PIC 13.3 μg/mL (a concentration that could theoretically be present in the media not taking into account degradation of the molecule over time), 4.4 μg/mL and 1.33 μg/mL (Fig. 7A).

Microglia phenotype was tested using several validated markers. Acknowledging that this is a simplification necessary to facilitate interpretation of the data, we used a nomenclature based on previous works^5,32^. We distinguished three phenotypes according to the mRNA expression levels of markers listed in brackets: immunomodulatory (*Il1rn*, Socs3), proinflammatory (*Nos2*, *Ptgs2*) and anti-inflammatory (*Lgals3*, *Igf1*).

*Socs3* and *Il1rn* mRNA levels in microglia were both specifically upregulated by CM from proliferating OPCs treated with PIC for 72 h compared to microglia maintained in control media (Fig. 7B–B’). Regarding *Il1rn*, although its mRNA level was upregulated over control also in microglia directly treated with PIC 13.3 μg/mL, the increase was significantly higher in microglia treated with CM from OPCs exposed to PIC for 24 h and 72 h in proliferation medium. CM from proliferating OPCs was also significantly more efficient in inducing *Il1rn* expression in microglia compared to that collected from differentiating OLs both at 24 and 72 h (Fig. 7B’).

The mRNA level of the proinflammatory marker *Nos2* in microglia was significantly upregulated only when treated with CM from differentiated cells (Fig. 7C). On the contrary, *Ptgs2* levels were induced by the two higher doses of PIC and by CM from both proliferating OPCs or differentiating OLs treated with PIC for 72 h (Fig. 7C’).

*Lgal3* was downregulated only in microglia treated with CM derived from OLs exposed to PIC for 24 h (Fig. 7D); whereas *Igf1* reduction was seen in microglia in all the concentrations of PIC tested and with all the conditional media from OLs exposed to PIC (Fig. 7D’).

*Ccl2* overexpression observed herein could play a role in recruiting microglia, therefore we measured the mRNA level of *Ccr2*. We showed that CM from proliferating OPCs, but not differentiating OLs, was able to upregulate the expression of *Ccr2* (Fig. 7E).

These data suggest that the two population of OLs differentially responded to TLR3 stimulation resulting in a different effect on microglia phenotype.

**Fig. 8:**
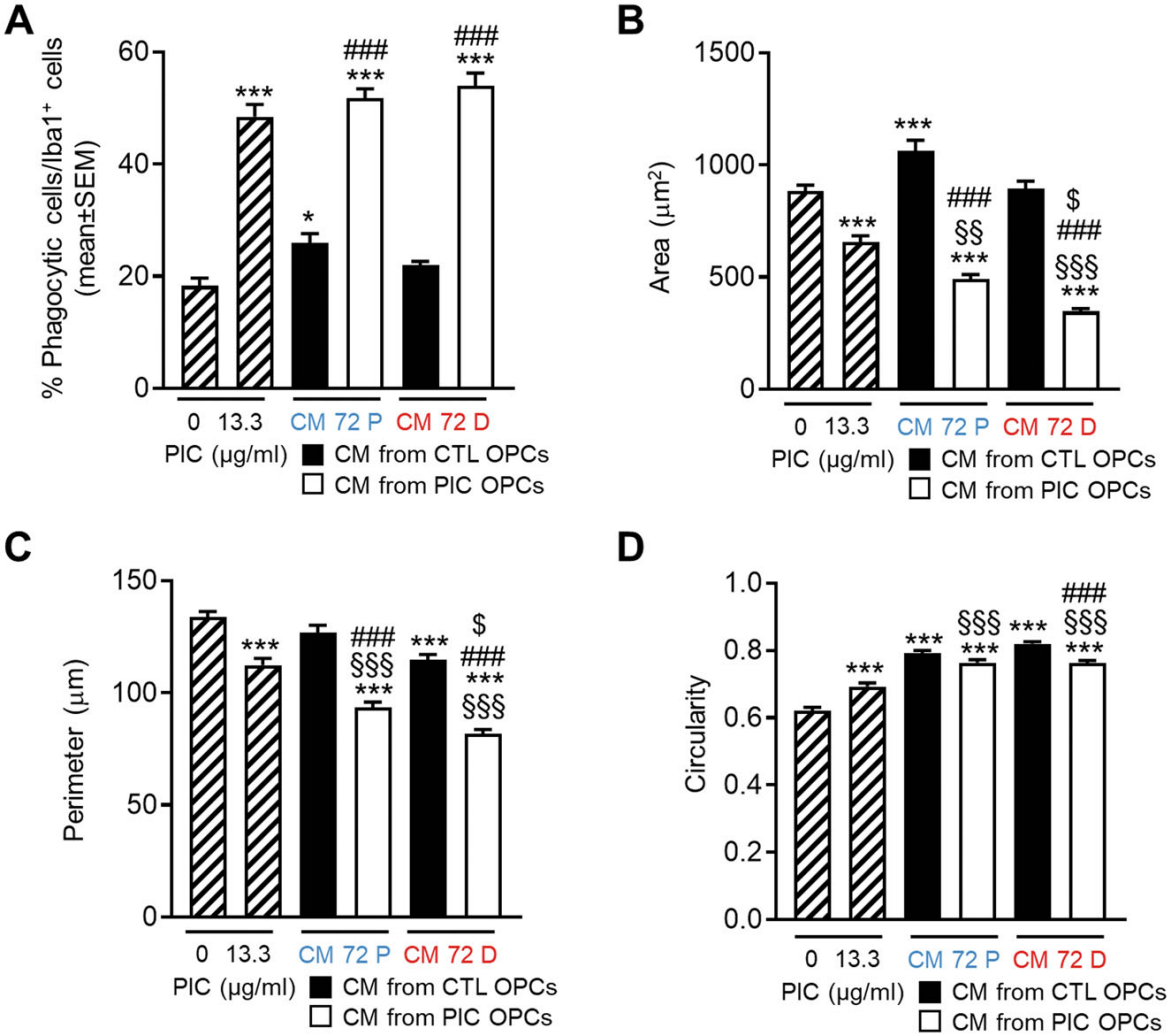
Conditional medium (CM) from proliferating and differentiating OLs regulates microglia phagocytosis and cell morphology. **A** Histograms reported the percentage of phagocytic cells determined as: number of cell with beads/number of total Iba1+ cells. Results are the mean of three independent experiments performed in triplicate ±SEM. **B**–**D** Microglial cell morphological changes induced by PIC or conditional medium considering cells area (**B**), cells perimeter (**C**), and cells circularity (**D**). Data are the mean of at least 250 cells/condition ±SEM. All comparisons were performed using One-way Anova followed by Bonferroni’s multiple comparison test. *P < 0.05 and ***P < 0.001 vs CTRL, ^###^*P* < 0,001 CM from CTRL OPCs vs CM from PIC OPCs, ^§§§^*P* < 0.001 and ^§§^*P* < 0.01 vs PIC, ^$^*P* < 0.05 PIC CM P vs PIC CM.

In light of the previously mentioned results, we explored whether primary microglia functions were altered by CM from either differentiated OLs or precursors kept in proliferating media in control condition or stimulated with PIC for 72 h (Fig. 8). First, phagocytosis was assessed treating the cells with specific fluorescent microbeads (Fig. 8A and Supplementary Fig. 3). We observed a significant increase in the percentage of microbeads phagocytosed per cell in microglia cultured in CM from proliferating OPCs and differentiating OLs treated with PIC both compared to cells maintained in control microglia media and to control CMs. In addition, microglia cultured in presence of PIC were also able to phagocyte microbeads. CM from unstimulated proliferating OPCs had a small effect on increasing phagocytosis capability of microglia.

Morphological analysis (Supplementary Fig. 4) showed a significant decrease in the cell body area (Fig. 8B) and perimeter (Fig. 8C) and an increase in circularity (Fig. 8D), all markers of microglia activation^24^, in microglia cultured in CM from both proliferating OPCs or differentiating OLs treated with PIC. Interestingly, direct challenge with PIC induced less morphology changes toward amoeboid shape compared to both CM derived from OLs treated with PIC. Finally, further confirming that the different response of the two population of OLs to TLR3 stimulation resulted in a different effect on microglia phenotype, both area and perimeter were more affected by CM from differentiating OPCs treated with PIC compared to proliferating OPCs treated with TLR3 agonist.

## Discussion

Using a well-established model of perinatal neuroinflammation^3,5^, we demonstrated a differential response to inflammation of PDGFRα+ OPC and O4+ immOL populations, revealing that cells already committed towards a more mature phenotype have greater inflammatory capacity. Although OLs are typically considered target of detrimental inflammation, recent studies indicate they may play an active and critical role in this process^28^. Indeed, following cuprizone-induced demyelination, adult OPCs upregulate IL-1β and CCL2 becoming ‘activated’ and reverting their transcriptome to resemble that of neonatal state^33^. Interestingly, the two proinflammatory mediators can act both in an autocrine fashion, stimulating OPCs migration, and in a paracrine way attracting microglia cells. Activation of TLR3 leading to an upregulation of IL-1β, CCL2, and CXCL10 are also seen in models of autism and schizophrenia associated with white matter abnormalities^2,34^. It would be important to see whether OLs play a role in these cases.

In accordance with studies in other cell types, we showed that the TLR3 agonist PIC can transcriptionally regulate TLR3 in OLs^35^. Further, we showed that OL stimulation with PIC leads to differential upregulation of *Ccl2* and *Cxcl10* transcripts based on the maturation state of the cells. This differential effect was specific to TLR3 stimulation and could not be recapitulated by IFNγ. Indeed, of the several chemokines that were surveyed, CCL2 was the most abundant chemokine that was present in the PIC stimulated CM, particularly in differentiating OLs. In agreement with previous results^36^, PIC led to incomplete differentiation of OLs and supported the hypothesis that TLR3 activation in the differentiating population in vivo could be one pathway by which OL maturation is blocked in the IL-1β model, since IL-1β per se does not block OL differentiation^37,38^.

A major finding is the demonstration that the maturation state of TLR3-stimulated OLs influences OLs-microglia signalling. *Socs3* and *Il1rn* mRNA were both specifically upregulated in microglia exposed to proliferating OL-CM. In microglia SOCS3 has a critical role in inhibiting cytokine signaling and activation of microglia through suppressing STAT3^39^. *Il1rn*, has been shown to be neuroprotective and result in the downregulation of the proinflammatory marker *Nos2*^40^. Here, we observed that differentiated OLs but not proliferating OPCs upregulated *Nos2* mRNA in microglia. Finally, as already observed in the cuprizone model of demyelination^33^, *Ccl2* production by undifferentiated OLs could play a role in recruiting microglia since *Ccr2* expression is modulated in microglia by IL-1β^19^ and CM from proliferating OLs. Thus the two populations of premyelinating OLs could affect microglial differentially. Further, OL-CM stimulated with PIC increased the phagocytic activity of microglia and morphologically these cells tended to be smaller and more rounded.

In conclusion, we showed that OLs regulate important genes associated with an innate immune response during perinatal inflammation in the IL-1β model of WMI in preterm infants. Our results suggest a possible mechanism by which IL-1β could induce a maturation block in O4+ OLs. Future studies are needed to elucidate the contribution of TLR3 and identity of the signaling pathway involved in OL maturation block in vivo.

## Supplementary information

Supplementary Data
