# Supplementary material for: The immune-inflammatory response of oligodendrocytes in a murine model of preterm white matter injury: the role of TLR3 activation
Source: Cell Death Dis. 2021 Feb 8;12(2):166. doi: 10.1038/s41419-021-03446-9 (PMC7870670; doi:10.1038/s41419-021-03446-9)
Supplement: Supplementary file 1 — Supplementary Data [file 41419_2021_3446_MOESM1_ESM.docx]

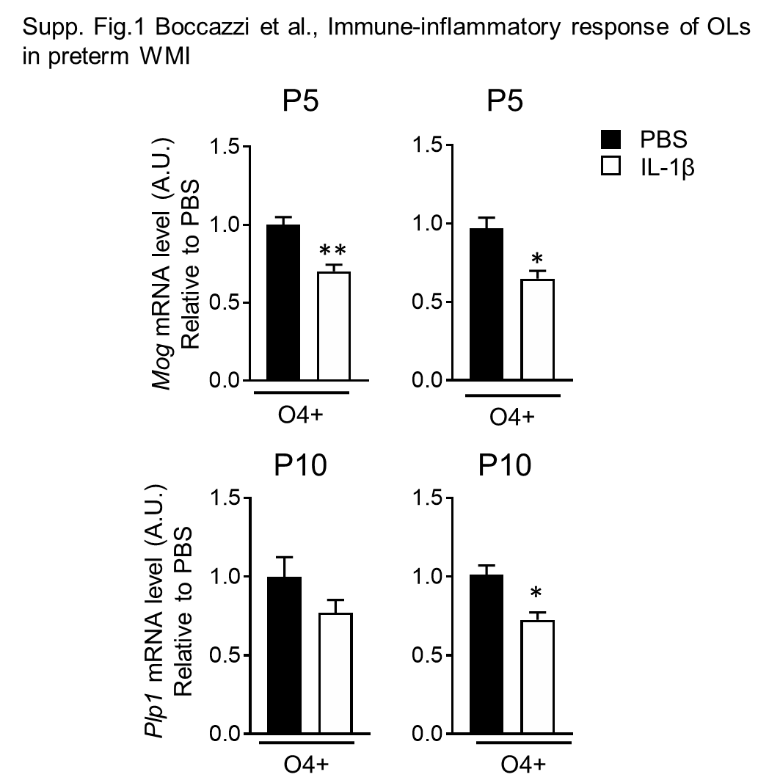
**Figure S1. IL-1β injections induce defects in the expression of typical myelin genes in O4+ cells.** *Mog* and *Plp* gene expression has been assessed by qPCR in the O4+ population sorted at P5 and P10 from control mice (data are mean ± SEM, n=18 group for each condition from 3 independent experiments, *P<0.05 and **p<0.01 by Unpaired T test).


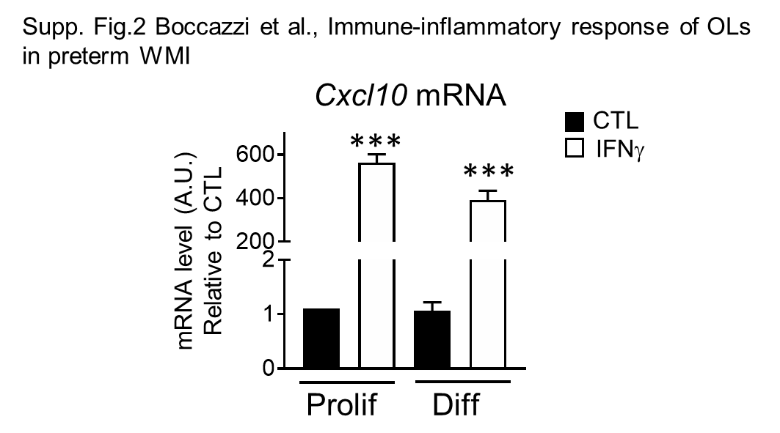


**Figure S2. IFNγ stimulation results in the upregulation of *Cxcl10* in both proliferating and differentiating OLs**. Primary OLs have been treated with IFNγ for 72 hours in proliferation (Prolif) or differentiation (Diff) medium. The expression of *Cxcl10* has been assessed by qPCR (data are mean ± SEM, n=3, ***P<0.001, Unpaired T test).

**Supplementary table 1. Gene name and primer sequences for primers for qRT-PCR.**

**
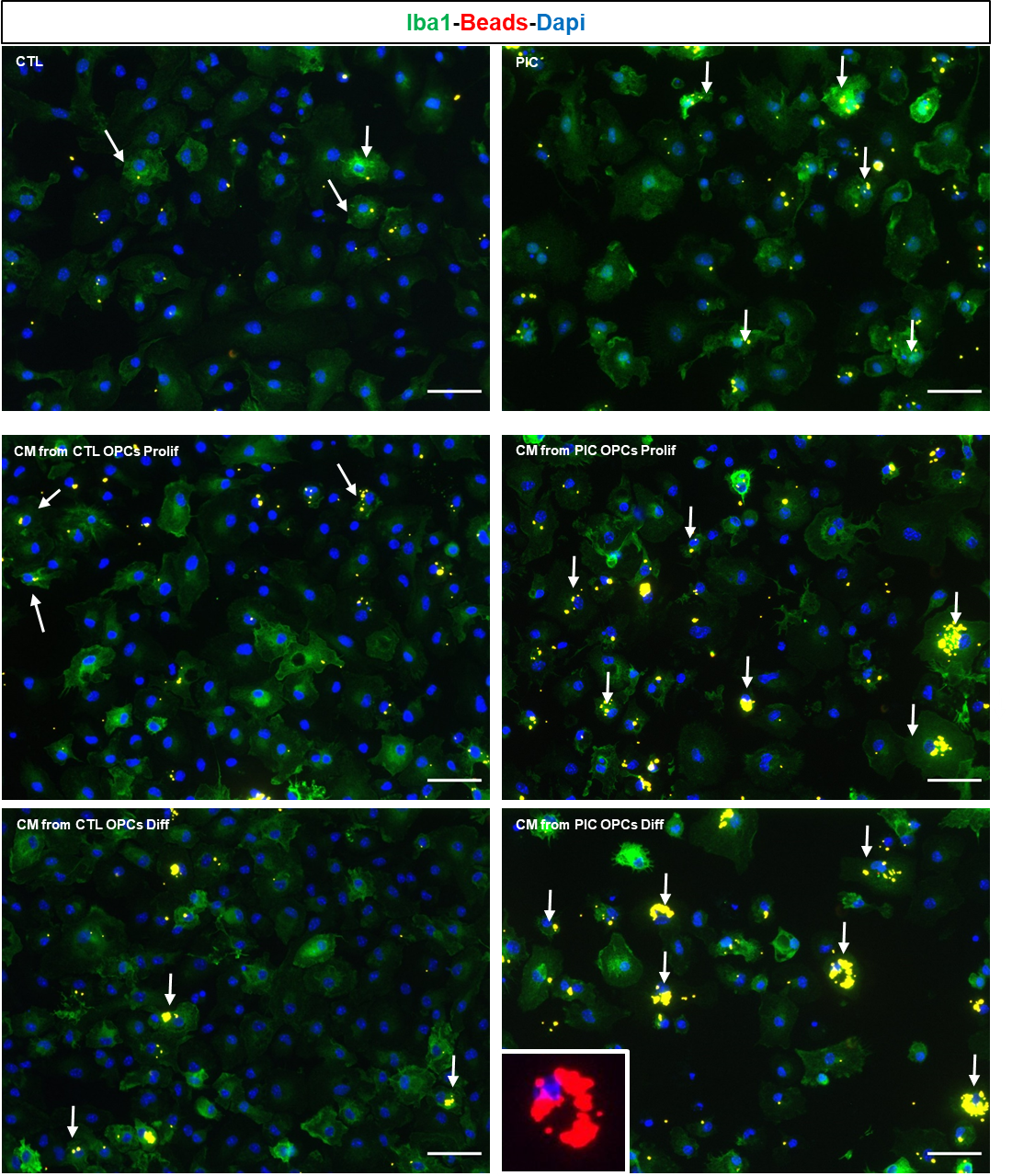
**

**Figure S3. Conditional medium (CM) from proliferating and differentiating OLs treated with Poly(I:C) increase microglia phagocytosis.** Representative photomicrographs in which the co-localization between red fluorescent microbeads and Iba1+ microglia cells is shown in yellow and pointed out with white arrowheads. Bar = 50 μm.


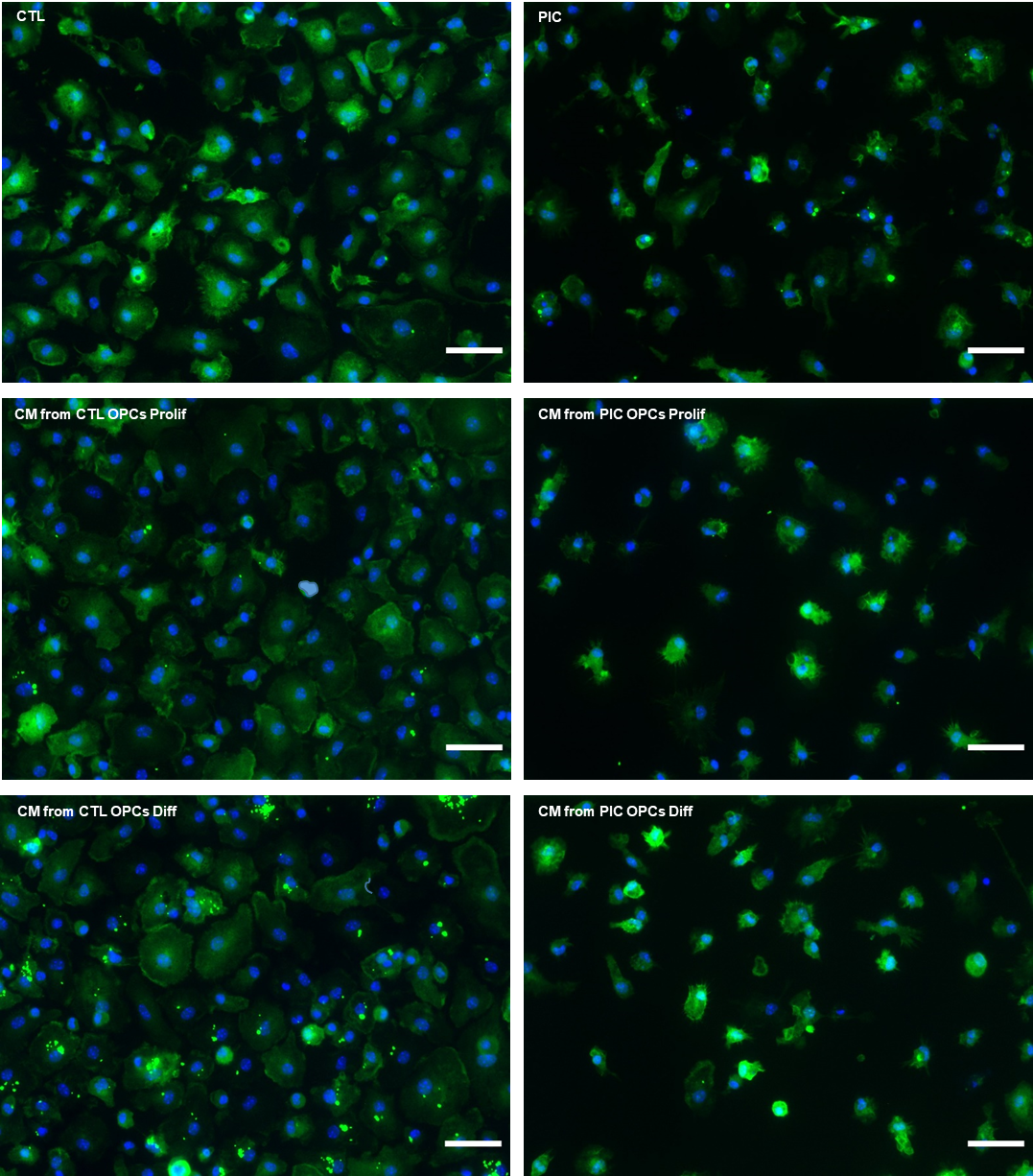


**Figure 4S. Microglial morphology changes upon exposure to conditional medium (CM) from proliferating and differentiating OLs treated with Poly(I:C).** Representative images of Iba1+ microglia cultured in microglia medium (±PIC) or in CM from OLs. Bar = 50 μm
